# Artery buckling affects the mechanical stress in atherosclerotic plaques

**DOI:** 10.1186/1475-925X-14-S1-S4

**Published:** 2015-01-09

**Authors:** Arnav Sanyal, Hai-Chao Han

**Affiliations:** 1 Department of Mechanical Engineering The University of Texas at San Antonio Biomedical Engineering Program, UTSA-UTHSCSA, TX, USA; 2 Department of Mechanical Engineering, The University of Texas at San Antonio, San Antonio, TX 78249, USA

## Abstract

**Background:**

Tortuous arteries are often seen in patients with hypertension and atherosclerosis. While the mechanical stress in atherosclerotic plaque under lumen pressure has been studied extensively, the mechanical stability of atherosclerotic arteries and subsequent effect on the plaque stress remain unknown. To this end, we investigated the buckling and post-buckling behavior of model stenotic coronary arteries with symmetric and asymmetric plaque.

**Methods:**

Buckling analysis for a model coronary artery with symmetric and asymmetric plaque was conducted using finite element analysis based on the dimensions and nonlinear anisotropic materials properties reported in the literature.

**Results:**

Artery with asymmetric plaque had lower critical buckling pressure compared to the artery with symmetric plaque and control artery. Buckling increased the peak stress in the plaque and led to the development of a high stress concentration in artery with asymmetric plaque. Stiffer calcified tissue and severe stenosis increased the critical buckling pressure of the artery with asymmetric plaque.

**Conclusions:**

Arteries with atherosclerotic plaques are prone to mechanical buckling which leads to a high stress concentration in the plaques that can possibly make the plaques prone to rupture.

## Background

The rupture of atherosclerotic plaques in coronary arteries leads to thrombus occlusion and heart attack. Similarly, the rupture of atherosclerotic plaques in carotid arteries leads to cerebral occlusion and strokes. Due to the severe consequences of plaque rupture and the risk in interventional treatment, it is of critical clinical importance to identify vulnerable plaques. Myriads of clinical and basic studies have demonstrated that the rupture of plaque depends on the morphology, components, and biological environment of the plaque [1-3]. Yet, the mechanism of plaque rupture has not been fully understood.

Extensive biomechanical studies have shown that mechanical stress concentrations in plaques play a critical role in the rupture of plaques [4-6]. The stress in the plaque is determined by the geometry of the plaque, the composition and structure of the plaque, as well as the mechanical loads including the lumen pressure and axial stretch [7-9]. However, the effect of mechanical instability i.e. artery buckling on plaque stress has not been investigated.

Atherosclerotic carotid arteries are often tortuous but the mechanism of their tortuosity remains unclear [10-12]. We have previously showed that arteries buckle under lumen pressure and axial tension making them tortuous, thereby affecting the wall stress distribution in the vessel wall [13-16]. It is important to understand the relationship between artery tortuosity and stress in atherosclerotic plaque. Previous work by Tang and colleagues has demonstrated that cyclic bending of coronary arteries can increase the peak stress in atherosclerotic plaque [17,18]. Therefore, it is necessary to study the buckling stability of atherosclerotic arteries and understand its possible effect on the mechanical stress in the plaques.

Accordingly, the objective of this study was to investigate the buckling behavior of arteries with atherosclerotic plaques and the subsequent effects on the mechanical stress in plaques.

## Methods

We performed a structural finite element analysis to simulate the buckling and post-buckling behavior of a coronary artery with symmetric and asymmetric (eccentric) atherosclerotic plaque. The effect of plaque on arterial stability was evaluated by comparing the critical buckling pressure and the maximum stress developed in the normal artery and the stenotic artery with plaque.

**Figure 1:**
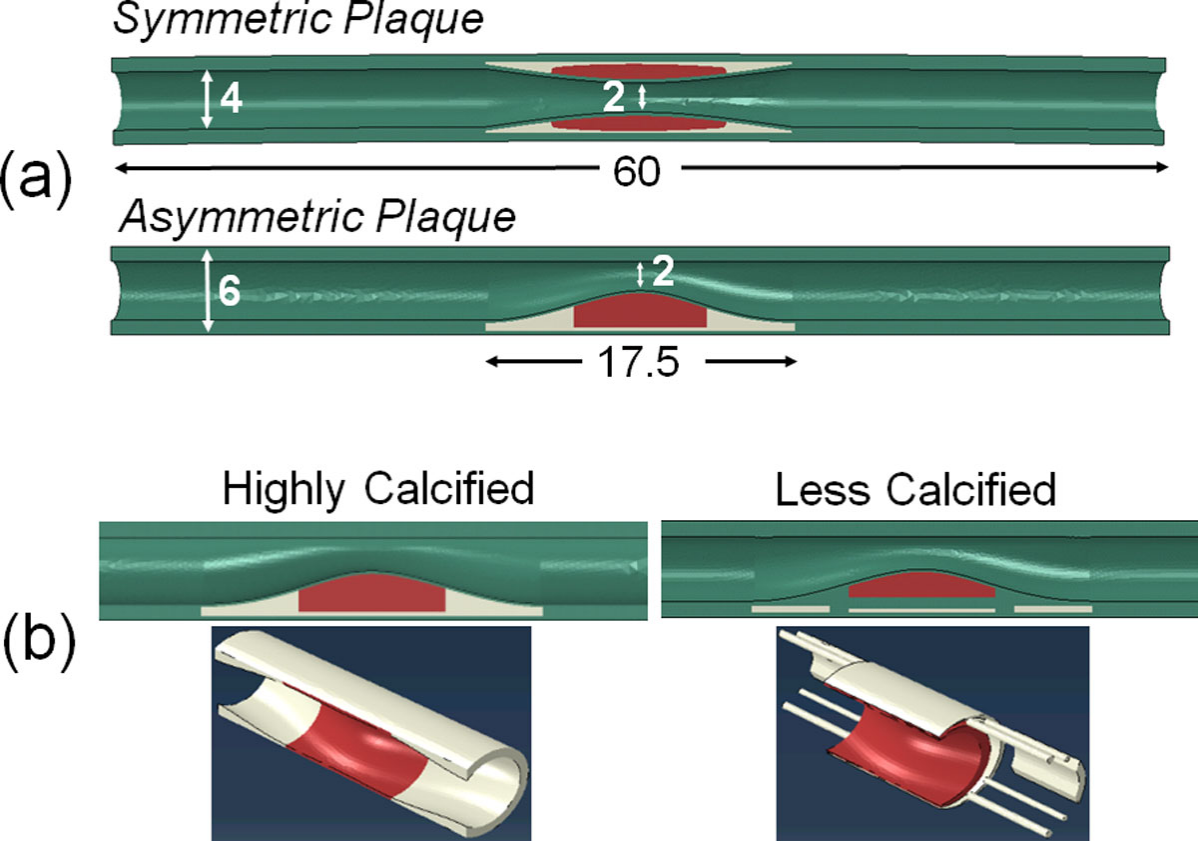
**(a) Schematic (longitudinal-section view) of a model coronary artery with a symmetric and an asymmetric plaque located at its center**. The green region represents the artery, red region is the lipid pool and white region is the calcification. All dimensions are in mm. (b) Longitudinal-sectional and three-dimensional views of an asymmetric plaque with different degrees of calcification. The left figure shows a fully calcified plaque, whereas the right figure shows a partially calcified plaque with a scattered distribution.

A cylindrical model of coronary artery was created using Solidworks^® ^(Dassault Systèmes, Waltham, MA). The unloaded vessel had a dimension of length 60 mm, outer diameter of 6 mm and wall thickness of 1 mm based on previous reports [17,19]. Atherosclerotic plaque models were created in the middle of the artery and the plaque geometry was either symmetric or asymmetric with respect to the artery axis (Figure 1(a)). The plaque was assumed to consist of a lipid pool and calcified tissue surrounded by a thin fibrous cap (cap thickness = 0.5 mm). The diameter of the throat of the plaque (2 mm) was half the diameter of the lumen (4 mm), resulting in a stenosis severity of 75% by cross-sectional area [20].

The arterial wall was assumed to behave as a homogenous, incompressible, orthotropic, nonlinear material with the Fung strain energy function of the form [21]:

$$W=\frac{b_{0}}{2}e^{Q}$$

(1) $$Q=b_{1}{E_{\theta }}^{2}+b_{2}{E_{z}}^{2}+b_{3}{E_{r}}^{2}+2b_{4}E_{\theta }E_{z}+2b_{5}E_{z}E_{r}+2b_{6}E_{\theta }E_{r}$$

where *b_0, _b_1_, b_2_, b_3_, b_4_, b_5_, b_6 _*are material constants from Wang et.al [22] (Table 1). The fibrous cap was also assumed to have the same material property as that of the artery. The lipid pool and calcified tissue were assumed to behave as incompressible, isotropic material with the neo-Hookean strain energy function. The stiffness of the lipid pool and the calcified tissue were assumed to be one-tenth and ten times the stiffness of the artery wall, respectively [17]:

**Table 1:** Material constants of the Fung's exponential strain energy function for the artery used in model simulations (from [20]).

b_0 _(kPa)	b_1_	b_2_	b_3_	b_4_	b_5_	b_6_
29.01	0.61	1.53	0.44	0.32	0.06	0.12

(2) $$\text{W}=\text{C}\left({\text{I}}_{1}\text{ - }3\right)$$

with C_lipid _= 0.1b_0 _and C_calcified-tissue _= 10b_0_

The buckling behavior of all arterial models (normal control, stenotic with symmetric plaque, or with asymmetric plaque) was simulated using the commercial FEA package ABAQUS^® ^(v6.11, Dassault Systèmes, Waltham, MA). The models were meshed using hybrid quadratic tetrahedral elements. Since coronary arteries are generally under very little axial pre-stretch [23,24], an axial displacement equivalent to a stretch ratio of 1.05 (i.e. 5% axial stretch) was applied to all nodes at the distal end of the arteries. A static internal pressure was applied to the lumen of the arterial models and the external pressure was set at zero. Both ends of the arteries were assumed as fixed with no lateral displacement or rotation, but were allowed to expand radially. These end conditions simulated the expansion of arteries under pressure *in vivo *and minimized the possible edge effects at the ends [25]. A small initial bend of 1 degree along the central axis of the normal artery model and the symmetric plaque model was created as an imperfection to facilitate the buckling analysis. The maximum lateral deflection of the central axis of artery was determined by averaging the deflections of two edges of the artery wall at the mid-point of the artery and was plotted against the lumen pressure. The pressure at which the deflection starts to increase from baseline and reaches a value of 0.5 mm was defined as the critical buckling pressure [15,26].

To determine the effect of buckling on maximum stress developed in the plaque, we compared two stenotic artery models with symmetric plaques, but different neck lengths (a long neck with length = 60 mm and a short neck with length = 30 mm). With the different neck lengths, the two models had different critical buckling pressures, such that at physiological pressures, the atherosclerotic vessel with long neck buckled while the control one with the short neck remained unbuckled. This approach allows us to specify the effect of buckling on plaque stress since the pressure and axial stretch ratio all remain the same in both models.

To determine the effect of amount of calcification and size of the lipid pool, an additional model of an asymmetric plaque was created with a smaller volume of the calcified tissue and lipid pool. The calcified tissue had a scattered distribution as shown in Figure 1(b).

A sensitivity analysis was performed to determine the effect of change of stiffness of plaque components on the buckling behavior of the asymmetric plaque model. The modulus of the lipid pool and calcified tissue (constant C in equation (2)) of the asymmetric plaque model was varied by ±50% from their baseline values [20]. Another analysis was performed to determine the effect of stenosis severity. The throat diameter of the asymmetric plaque model was changed to 1.2 mm and 2.8 mm resulting in stenosis severity of 90% (more stenosis) and 50% (less stenosis) by cross-sectional area, respectively. In all these simulations, the boundary conditions were kept the same as described above and the deflections were determined for increasing lumen pressures. The deflections were then plotted with the pressure and the critical buckling pressure was determined as described above.

## Results

**Figure 2:**
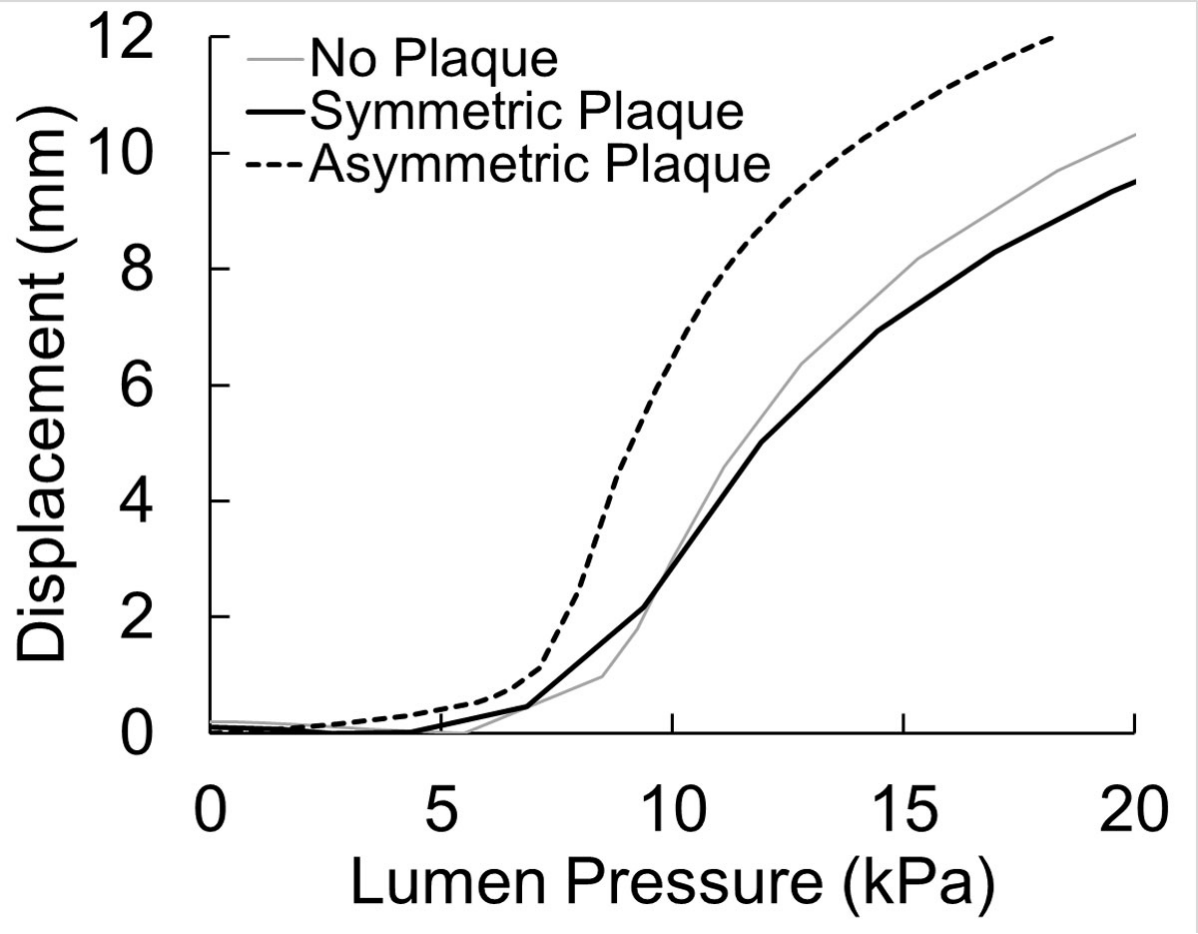
**Buckling displacement (maximum deflection at the middle) plotted as function of lumen pressure of a normal artery, artery with symmetric plaque, and artery with an asymmetric plaque**.

The presence of an atherosclerotic plaque affects the critical buckling pressure of a coronary artery. The buckling behavior of the stenotic artery with plaque was different from the normal artery and different for symmetric and asymmetric plaques. The stenotic artery with a symmetric plaque had a critical buckling pressure (7.0 kPa) close to that of a normal artery (6.9 kPa). However, the stenotic artery with an asymmetric plaque had a much lower critical buckling pressure (5.5 kPa) (Figure 2). The post-buckling deflection increased rapidly for the stenotic artery with asymmetric plaque compared to normal artery, whereas stenotic artery with symmetric plaque had a slightly slower rise in the deflection.

**Figure 3:**
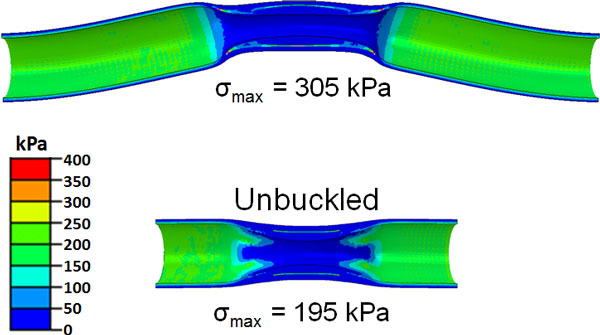
**von Mises stress profile in buckled and unbuckled artery with a symmetric plaque under the same lumen pressure of 100 mmHg (13.33 kPa)**. The neck was cut short in the bottom model to avoid buckling.

**Figure 4:**
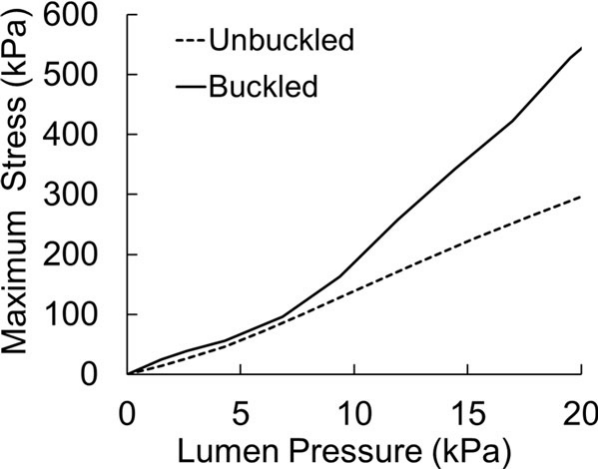
**Comparison of peak von Mises stress as a function of lumen pressure for a buckled and an unbuckled artery with symmetric plaque**.

For the artery with a symmetric plaque, the maximum stress developed in the buckled artery was larger than that of the unbuckled artery (Figure 3). The stress in the plaque for the buckled artery increased much more significantly with an increase in the lumen pressure (Figure 4).

**Figure 5:**
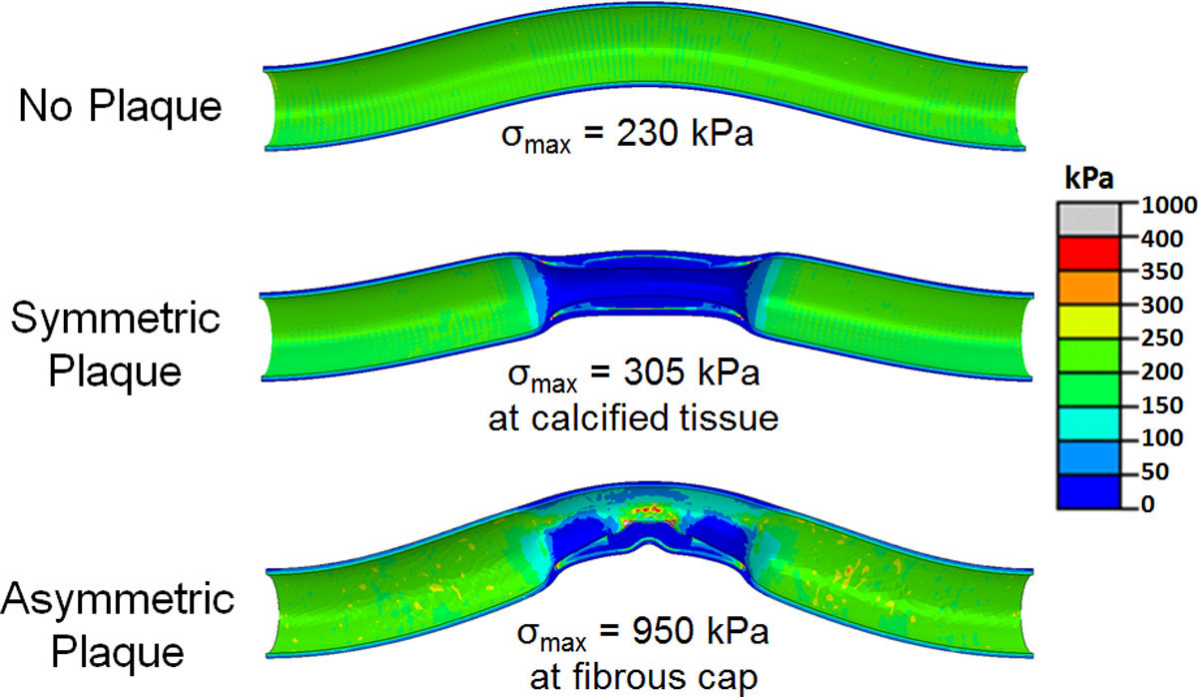
**von Mises stress distribution in the artery lumen and the plaque (longitudinal-section view) for a normal artery, artery with symmetric plaque and artery with asymmetric plaque at a lumen pressure of 100 mmHg**.

**Figure 6:**
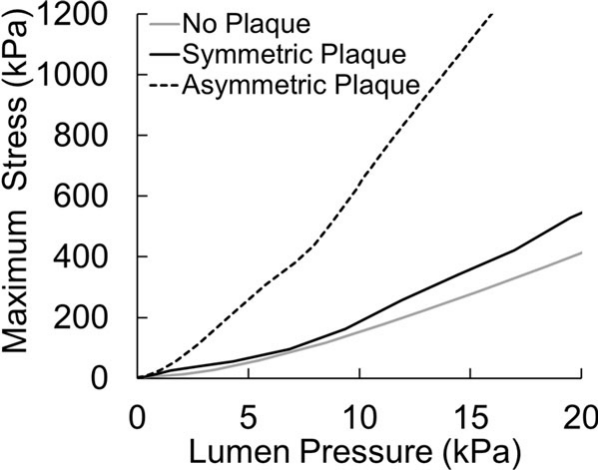
**Peak von Mises stress plotted as a function of lumen pressure for a normal artery and stenotic artery with symmetric and asymmetric plaques**.

The symmetry of the plaque (symmetric or asymmetric) had a significant effect on the location and magnitude of peak stress in the plaque post-buckling (Figure 5). At a physiological lumen pressure of 100 mmHg, the maximum stress in the symmetric plaque model was observed in the calcified tissue and its magnitude was 32% higher than the maximum stress in the lumen of the normal artery (305 kPa vs 234 kPa). However, the maximum stress in the asymmetric model was observed in the fibrous cap located slightly away from the center of the artery and had almost four-fold higher magnitude (960 kPa). Also, the maximum stress in the asymmetric plaque model increased much more rapidly with increasing lumen pressure compared to the symmetric plaque model (Figure 6).

**Figure 7:**
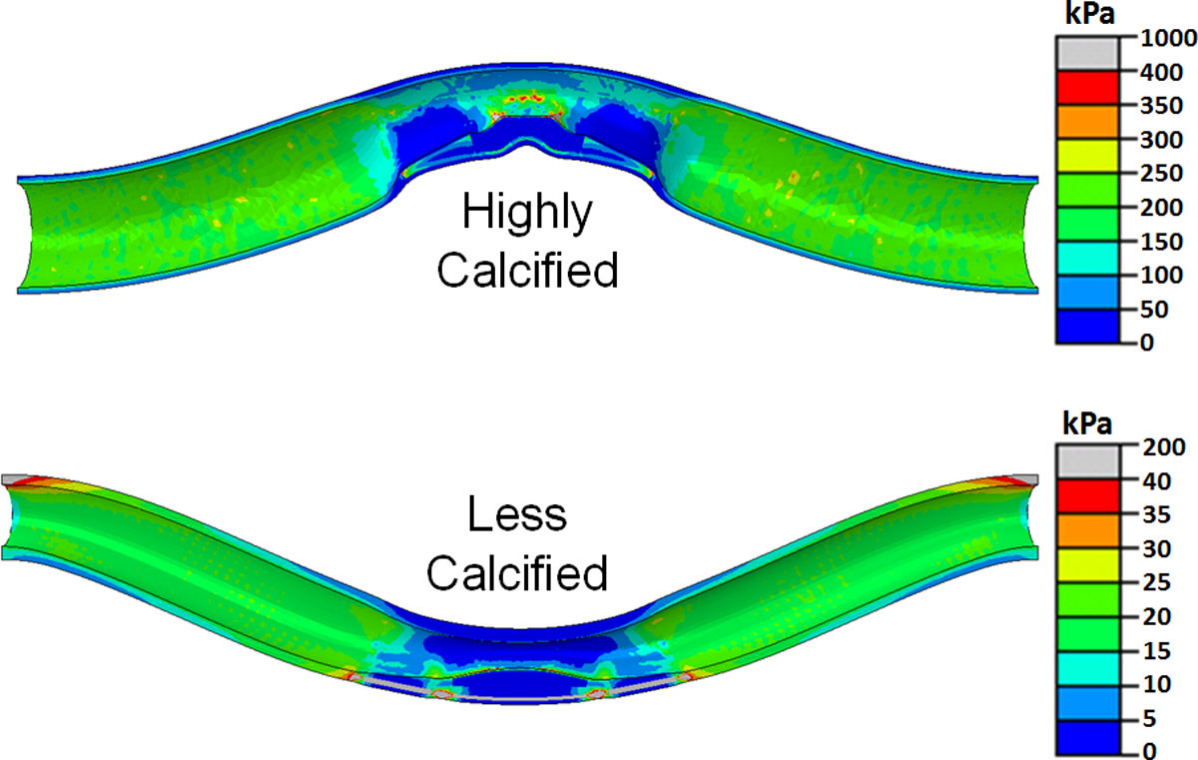
**Deformed shape and stress distribution in the plaque and lumen of an artery with asymmetric plaques with different degrees of calcification**. The maximum deflection in both cases is around 11 mm but at different lumen pressures (see Figure 8). Note the ten-fold difference in the scales of the two stress maps.

**Figure 8:**
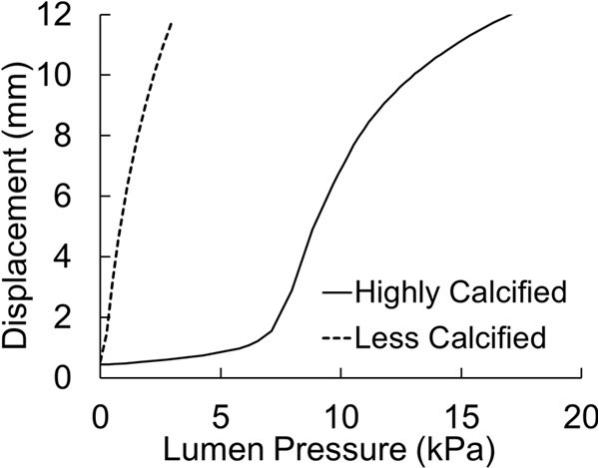
**Buckling deflections plotted as functions of lumen pressure of arteries with asymmetric plaque with different degrees of calcification**.

An interesting observation was that for asymmetric plaque of major lipid pool and major calcification cores, the buckling direction could be different and thus the stress pattern/distribution could be different. A less calcified asymmetric plaque deflects almost immediately after pressure is applied (near-zero critical buckling pressure) and the deflection is towards the side of the plaque. In contrast, a highly calcified asymmetric plaque buckles at a certain critical buckling pressure. Moreover, the deflection is towards the opposite side of the plaque (Figures 7 &8).

**Figure 9:**
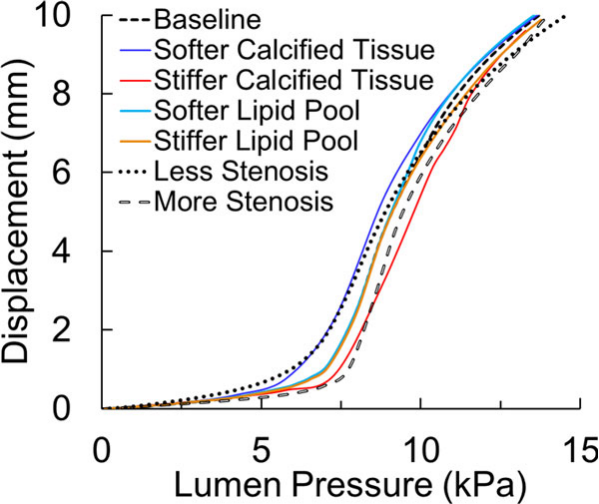
**Buckling deflection plotted as a function of lumen pressure for the artery with an asymmetric plaque (baseline model) and its several variations of stiffness of calcified tissue, stiffness of lipid pool and stenosis severity**. The baseline model had material properties as per equation (2) and had a stenosis severity of 75% by cross-sectional area.

For the artery with asymmetric plaque, the change in stiffness of the lipid pool by ±50% had negligible effect on both the critical buckling pressure and the maximum stress in the fibrous cap at 100 mmHg lumen pressure (Figure 9). However, a 50% increase in the stiffness of calcified tissue resulted in 7% higher buckling pressure (5.9 kPa) and 4% lower stress in the fibrous cap (922 kPa). Similarly, a 50% decrease in the stiffness of the calcified tissue resulted in 13% lower critical buckling pressure (4.8 kPa) and a 2.5% higher stress in fibrous cap (983 kPa).

The reduction of stenosis severity from 75% to 50% in terms of cross-sectional area resulted in 22% lower critical pressure (4.4 kPa) and a 60% reduction in the stress at the fibrous cap (382 kPa). However, increasing the stenosis severity to 90% resulted in 20% higher critical pressure (6.6 kPa) but only 3% decrease in stress at the fibrous cap (929 kPa) (Figure 9).

## Discussion

The results of this study show that stenotic arteries with atherosclerotic plaques are prone to mechanical buckling which can make them tortuous and lead to high stresses in the plaque. In addition, buckling leads to a faster rise in plaque stress with increasing pressure. The amount of calcification alters the local rigidity of the plaque and therefore affects the buckling deflection pattern and stress distribution in the plaque and artery. The change in stiffness of the calcified tissue and the stenosis severity has a noticeable effect on the buckling behavior and stress distribution in atherosclerotic plaque.

Our results are consistent with results from previous studies. Most plaques are eccentric and their rupture is most likely due to failure of the thin fibrous cap [3] which is consistent with our current simulation results. Tang et al. [17,18] demonstrated that cyclic bending had a significant effect on the development of high stress in the plaque, which is consistent with our observation that bent buckling of an artery results in a rapid rise in plaque stress. Our previous study on buckling of arteries with fusiform aneurysms also showed the significance of bent buckling in development of tortuosity and high stress concentration in aneurysm wall [27,28]. Recently, Abdelali et al. [29] demonstrated that the thin fibrous cap of atherosclerotic cap itself can buckle locally into a wavy shape. Our simulation of asymmetric plaque shows similar deformed shape with local buckling of the cap of the lipid pool. Furthermore, we also showed that there is a global buckling pattern of the whole artery segment, which leads to high stresses in the fibrous cap. Therefore, our results suggest that mechanical buckling makes atherosclerotic arteries tortuous and is an important factor that affects the plaque stress, which may lead to plaque rupture. In addition, Weinbaum and colleagues have recently demonstrated that scattered calcium cores can lead to high stress concentrations which can cause plaque rupture [30-32]. Similarly, our simulations showed that the scattered calcium cores also affect the buckling of artery with plaques and can alter the stress distribution in the plaque.

One limitation of this study was that we considered an idealized geometric model of the coronary artery and the plaque as well as simplified boundary conditions. The initial curvature of the artery was not considered and the arterial wall was assumed as homogenous. A previous study has shown that the effect of initial curvature on artery buckling is small [33]. In addition, the surrounding tissue support was not considered and the two ends of the artery were assumed to be fixed. These are idealized representation of the limited support available for coronary arteries in vivo since these arteries align on the surface of the myocardial wall. Changing the boundary constraints may lead to higher critical buckling pressures and different bucking patterns [14,34] which may affect the local curvature of the plaque and therefore the stress levels in the plaque. Secondly, the stress and buckling analysis done under static pressure. Previous studies have demonstrated that the buckling peak pressure and stress follow similar trends as demonstrated under static pressure, though the stress will be cyclic during a cardiac cycle [26,35,36]. The severity of stenosis will also affect the blood flow in the throat of the plaque with a local reduction in pressure. Such effects can be studied in the future using computational models with fluid-structure interaction [5,17]. In addition, the variations of lipid pool size and fibrous cap thickness, which may change the magnitude of the maximum stress and stress distribution in the plaque were not examined and needs further investigation. Despite these limitations, this study shows the susceptibility of stenotic artery to buckling which can lead to a higher peak stress.

The development of high peak stress in atherosclerotic plaque has been considered as a trigger for plaque rupture [4-6]. Thus, our simulation results suggest that the presence of an asymmetric-shaped or eccentric plaque can make artery more susceptible to rupture due to the development of high stress concentration in the fibrous cap of the plaque.

## Conclusion

Arteries with atherosclerotic plaques are prone to mechanical buckling which leads to a high stress concentration in the plaques that could possibly make the plaques prone to rupture. Mechanical buckling of the atherosclerotic plaque and the arterial wall could be a new factor that needs to be considered in studying the biomechanics of plaque rupture.

## Competing interests

The authors declare that they have no competing interests.

## Authors' contributions

HCH: study design, data analysis, and manuscript preparation, AS: finite element analysis, data analysis and manuscript preparation. All authors read and approved the final manuscript.

## Declaration

Publication of this article was paid with funding from grant HL095852 from the National Institutes of Health.

This article has been published as part of *BioMedical Engineering OnLine *Volume 14 Supplement 1, 2015: Cardiovascular Disease and Vulnerable Plaque Biomechanics. The full contents of the supplement are available online at http://www.biomedical-engineering-online.com/supplements/14/S1
